# An improved model framework linking the extracellular environment to antibody glycosylation

**DOI:** 10.1186/1753-6561-9-S9-P28

**Published:** 2015-12-14

**Authors:** Philip M Jedrzejewski, Karen M Polizzi, Cleo Kontoravdi

**Affiliations:** 1 Department of Chemical Engineering, Imperial College London, London SW7 2AZ, UK; 2 Department of Life Sciences, Imperial College London, London SW7 2AZ, UK; 3 Centre for Synthetic Biology and Innovation, Imperial College London, London SW7 2AZ, UK

## Background

Glycoproteins make up the bulk of biologically-derived medicines, and are taking up an ever increasing share of the prescription pharmaceuticals market. As opposed to small molecule drugs, glycoproteins are large complex molecules with heterogeneity arising from a multitude of glycan moieties. Glycans are complex post-translation modifications, which result from a number of enzymatic reactions in the ER and Golgi collectively known as glycosylation and play an important role in pharmacokinetics such as drug safety, efficacy and half-life. It is known that the availability of the nucleotide sugar donors (NSDs), which are the co-substrates to the enzymatic glycosylation reactions of the Golgi, can be affected by a number of process conditions such as culture mode, temperature, dissolved oxygen and nutrient availability, as well as the addition of precursor molecules to the culture medium [1]. Consequently, feeding strategies of nucleotide and nucleotide sugar precursors have been explored to exert control over the glycoform [2,3]. In this work, a mathematical model platform is presented to quantify the impact of nutrient availability and feeding strategies on the glycosylation process with the aim to enable the design of feeding strategies to optimise the product glycoform.

## Model development

As part of this work the Jedrzejewski et al. modelling platform was developed and trained further for improved model confidence and performance [4]. The framework links the extracellular environment, through the availability of intracellular metabolites in the cytoplasm and the Golgi apparatus, to the glycosylation of the conserved glycan site of the IgG heavy chain. The model platform comprises four parts, which are interlinked through dynamic fluxes and metabolite concentrations:

• A modified cell growth model based on Monod kinetics capturing cell culture dynamics and the impact of various hexose and nucleotide precursor additions to the cell culture media;

• A semi-structured purine and pyrimidine synthesis network describing the intracellular concentrations of nucleotide triphosphates, which are the co-substrates of NSD synthesis;

• A structured and mechanistic representation of the NSD synthesis pathway;

• The del Val et al. model describing the N-linked glycosylation process of the conserved glycan structure of the IgG antibody heavy chain [5].

The main focus of the work has been on the bottom-up mechanistic in silico reconstruction of the NSD synthesis network. The 34 species that make up the metabolic network were represented by means of mass balances that are connected through a network of 60 reactions, which were modelled as saturation rate kinetics based on reaction mechanisms found in the literature. The model platform is able to reproduce cell growth dynamics, extracellular nutrient availability, dynamic intracellular NSD and nucleotide concentrations, product titer and the antibody product glycoform.

## Results

**Figure 1:**
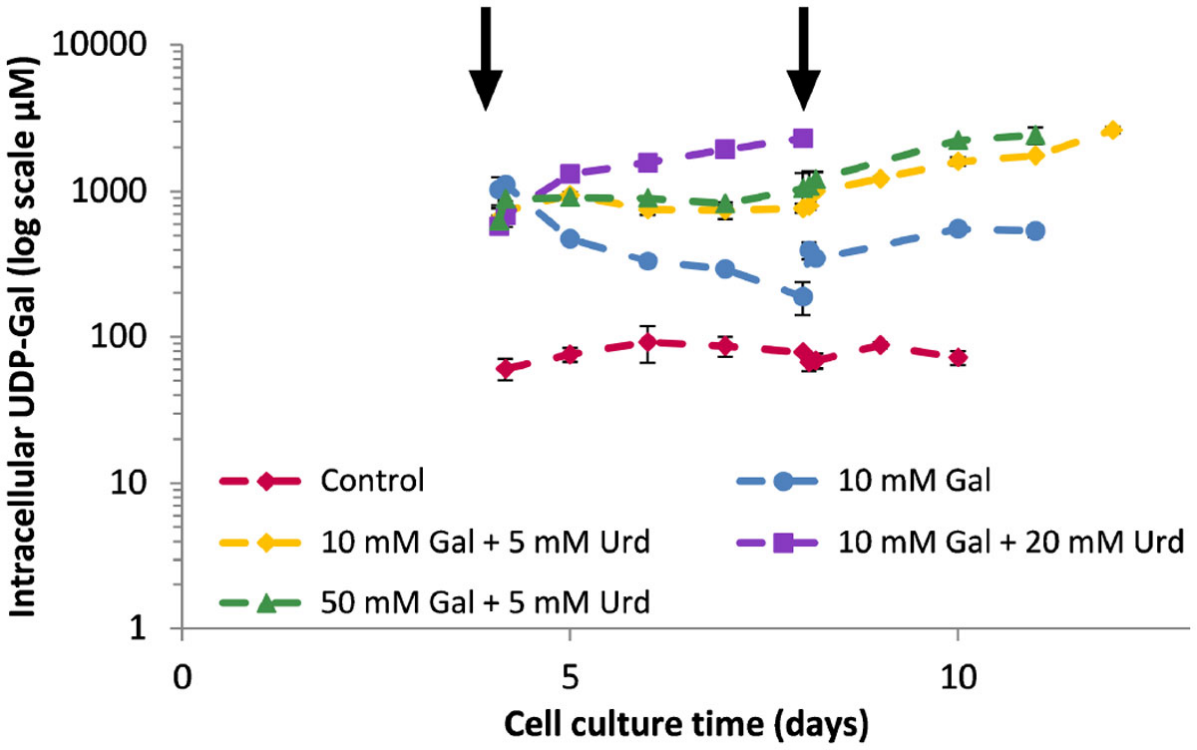
**Intracellular UDP-Gal concentrations for all five conditions of the 2^nd ^set of experiments with respect to culture time**. Feeding of galactose and/or uridine on days 4 and 8 of culture is indicated by the overhead arrows.

Refinement of the original Jedrzejewski et al. model framework and further training was achieved through a two-step CHO cell-based experimental process. All cultures were grown on the CD-CHO and CD EfficientFeedTM C platform (Life Technologies, Paisley, U.K.). In a first set of experiments, the experimental aim was to elicit a dynamic response of the NSD species GDP-Fuc and GDP-Man, to increase model confidence. The cells were fed with 10 mM mannose and 1 mM or 0.25 mM guanosine on day 5 of cell culture. Exploration of this part of metabolism was particularly important for the mechanistic representation of the NSD synthesis network, where inhibitory and control mechanisms across the network structure play an important role. In addition, the feeding of the nucleotide precursor guanosine was able to perturb and probe guanosine triphosphate (GTP) synthesis. The data allowed a model extension to fed-batch operation and including the impact of mannose and uridine additions on cell culture dynamics and nucleotide synthesis. A second set of experiments aimed at probing the metabolic factors affecting antibody galactosylation. In this experiment all CHO cell cultures were grown in manganese chloride-supplemented media and varying amounts of galactose and uridine were fed on days 4 and 8 of culture. The effects were dynamic perturbations with respect to cell culture dynamics, nucleotide and NSD synthesis as well as the antibody product glycoform. Most importantly, the effect of galactose and uridine addition on intracellular uridine diphosphate galactose (UDP-Gal) concentration (Figure 1) and the resulting increase in antibody product galactosylation were observed. An increase of up to 40% in galactosylation in the terminal product glycoform was observed as compared to the control cultures. Dynamic perturbations of intracellular uridine triphosphate were also observed and enabled further refinement of the nucleotide synthesis model structure.

## Conclusions

We have developed a mathematical model that links the extracellular environment to protein product glycoform for CHO cell cultures grown under batch and fed-batch conditions. The model output is in good agreement with data from a variety of culture conditions and is able to capture the dynamic impact of hexose and nucleotide precursor additions to culture media and their impact on NSD concentrations and product glycoform. Data from an experiment in which galactose and uridine were added to the cell culture media, in particular, extended the dynamic range of the model platform to regimes known to increase antibody galactosylation. Following this extensive model development and training exercise, the platform can now be used as an in silico tool towards designing feeding strategies to alter and drive the galactosylation of mAbs. Lastly, the modular nature of the framework allows it to be coupled with other models to translate the framework to other expression systems, operation modes and culture conditions.
